# Direct and culture-enriched 16S rRNA sequencing of cecal content of healthy horses and horses with typhlocolitis

**DOI:** 10.1371/journal.pone.0284193

**Published:** 2023-04-13

**Authors:** Luiza S. Zakia, Diego E. Gomez, Benjamin B. Caddey, Patrick Boerlin, Michael G. Surette, Luis G. Arroyo

**Affiliations:** 1 Department of Clinical Studies, Ontario Veterinary College, University of Guelph, Guelph, Ontario, Canada; 2 Department of Production Animal Health, Faculty of Veterinary Medicine, University of Calgary, Calgary, Alberta, Canada; 3 Department of Pathobiology, Ontario Veterinary College, University of Guelph, Guelph, Ontario, Canada; 4 Department of Biochemistry and Biomedical Sciences, McMaster University, Hamilton, Ontario, Canada; University of Illinois, UNITED STATES

## Abstract

Next generation sequencing has demonstrated that alpha diversity of the fecal microbiota is significantly altered in horses with typhlocolitis. The objective of this study was to evaluate the bacterial composition of the cecum content of horses with and without typhlocolitis through direct and culture-enriched 16S gene sequencing of six healthy horses and six horses with acute typhlocolitis; a case-control study design. Cecal content was collected after euthanasia. An aliquot was used for direct 16S gene sequencing. Another was serially diluted with brain heart infusion (BHI) and plated onto five different agar media. All culture medias, except for MacConkey, were incubated anaerobically. Bacterial colonies were harvested in bulk and used for DNA extraction, 16S PCR amplification, and sequenced using the Illumina MiSeq platform. Predominant phyla in healthy and diseased horses were *Firmicutes*, followed by *Bacteroidetes* in all cultured medias, except for MacConkey agar, in which *Proteobacteria* was the dominant phylum. Greater bacterial richness was identified in sequenced cecal contents as compared to cultured plates (P < 0.05). Culture-enriched molecular profiling combined with 16S rRNA gene sequencing offer an alternative method for the study of the gut microbiota of horses. For direct cecum content 16S gene amplification, the alpha diversity indices were lower in diarrheic horses compared to healthy horses (P < 0.05). A higher relative abundance of *Fusobacteriota* was found in 2/6 samples from diarrheic horses. The role of *Fusobacteriota* in equine colitis deserves investigation.

## Introduction

Acute equine typhlocolitis is associated with high morbidity and mortality in horses [1]. The risk factors for disease development are multifactorial, and encompass many etiological agents, inflammation, decreased microbial diversity and alteration of the gastrointestinal tract (GIT) microbial communities [2]. Currently, in approximately 60% of the cases of colitis, an etiological diagnosis cannot be reached [1, 3], likely due to both lack of consistency in diagnostic testing, but mostly due to the limited knowledge of the GIT microbiota of horses.

Currently, there is no gold standard for the evaluation of the gastrointestinal microbiota [4]. At present, the most commonly used methods for assessing the GIT microbiota are non-culture-based methods, such as quantitative real-time PCR and sequencing of 16S rRNA gene [5]. Bacterial culture offers a narrow view of the microbial communities of the GIT but paired with targeted (e.g., 16S rRNA gene sequencing) or untargeted (e.g., metagenomics) molecular methods its applicability can be expanded [4]. Over the past decade, microbiota studies in several species, including horses, have exploded exponentially. A few comprehensive reviews have been recently published [6–9], and summarize the current knowledge on equine GIT microbiota. Although many studies have been performed on this subject, the physiological and pathological microbial changes that occur in the gastrointestinal system, and their relationship with disease, are not yet fully elucidated. Specifically, the changes in the microbiota of horses with typhlocolitis require further studies.

The cecum and colon are the most important compartments for fermentation and energy production of the equine gastrointestinal tract and are highly susceptible to alteration of the microenvironmental populations [10]. The microbiological events that take place in these compartments in horses suffering from enterocolitis are largely unknown. Two studies comparing samples from healthy horses and horses with typhlocolitis have demonstrated a decrease in alpha diversity and a change in microbial community in diseased animals [11, 12]. However, Costa *et al*. [11] evaluated fecal samples, meaning their findings may not be representative of the cecum and colon of the cases of typhlocolitis. Arroyo *et al*. [12] did evaluate cecal and large colon samples, and compared healthy and colitis horses, as well as luminal versus mucosal samples. Although the results of these studies greatly contribute to our current knowledge, they are both limited to the target amplicon gene sequencing. Therefore, the aim of this study is to evaluate the microbiota composition of the cecum of healthy horses and horses with typhlocolitis using culture-enriched 16S rRNA gene sequencing. An additional objective of this study was to compare the culture-enriched sequencing results to direct 16S rRNA gene sequencing.

## Material and methods

### Animals and sample collection

Six healthy horses and six horses with typhlocolitis all > 1-year-old were included in this study. Healthy horses had no signs of gastrointestinal disease such as abdominal discomfort, loose manure and fever during the 4 weeks before enrollment and were presented to the Ontario Veterinary College (OVC) for euthanasia due to problems unrelated to the gastrointestinal system. None of the healthy horses had received anti-inflammatory or antimicrobial drugs within the previous 30 days. Diarrheic horses were horses hospitalized at OVC for treatment of typhlocolitis admitted with diarrhea and/or fever and that had cecal/colonic inflammation confirmed upon necropsy examination. To the best of our knowledge, these horses had not received antimicrobials prior to sample collection. Immediately after euthanasia, the horse was placed in left lateral recumbency. Then, an ultrasound guided puncture (14G 5.25 inches catheter, BD Angiocath, Sandy, Utah, United States) of the cecum was performed after the skin was aseptically prepared. Approximately, 10 ml of cecal content were obtained from each horse. The samples were immediately stored at -80°C until processing.

### Direct 16S amplicon sequencing samples (cecum samples)

All samples were thawed at room temperature and homogenized by pipetting. A total of 150μL were placed in a cryotube containing 1 ml of GES prepared using 60 g guanidine thiocyanate, 20 ml 0.5M EDTA (pH 8) and 20ml sterile ddH_2_O. The samples were kept at -20°C until bacterial culture was finished. All samples underwent DNA extraction and PCR at the same time.

### Bacterial culture

All samples were thawed at room temperature and homogenized by pipetting before being platted. Then, a 100μL aliquot of the cecal content was placed into a tube containing 900μL (10^−1^) of brain heart infusion (BHI) with 0.1 mM of L-cysteine hydrochloride. Samples were serially diluted to 10^−4^ then 100 μL of the 10^−2^ and 10^−4^ were inoculated onto the following culture media plates: fastidious anaerobic agar (Thermo Fisher Scientific, Mississauga, Ontario, Canada) with 5% horse’s blood (FAA), Columbia blood agar (Thermo Fisher Scientific, Mississauga, Ontario, Canada) with 5% of horse’s blood (CBA+HB), Columbia blood agar with 5% of rumen fluid added (CBA+RF), and BHI (Thermo Fisher Scientific, Mississauga, Ontario, Canada) with the addition of 0.5 g/L of L-cysteine, 10 mg/L of hemin, and 1mg/L of vitamin K (BHI). An aliquot of 10^−1^ and 10^−3^ dilutions were also inoculated onto MacConkey agar (Mac) (Thermo Fisher Scientific, Mississauga, Ontario, Canada) plates. A previous pilot study showed that this medium and culture conditions yielded fewer bacteria colonies than plates incubated under anaerobic conditions. Additionally, another 100μL of each non-diluted sample was placed in a tube with 100μL of absolute ethanol and incubated for 1 hour at room temperature. The 200μL were then platted on Columbia blood agar with 5% of horse blood (AS).

**Fig 1:**
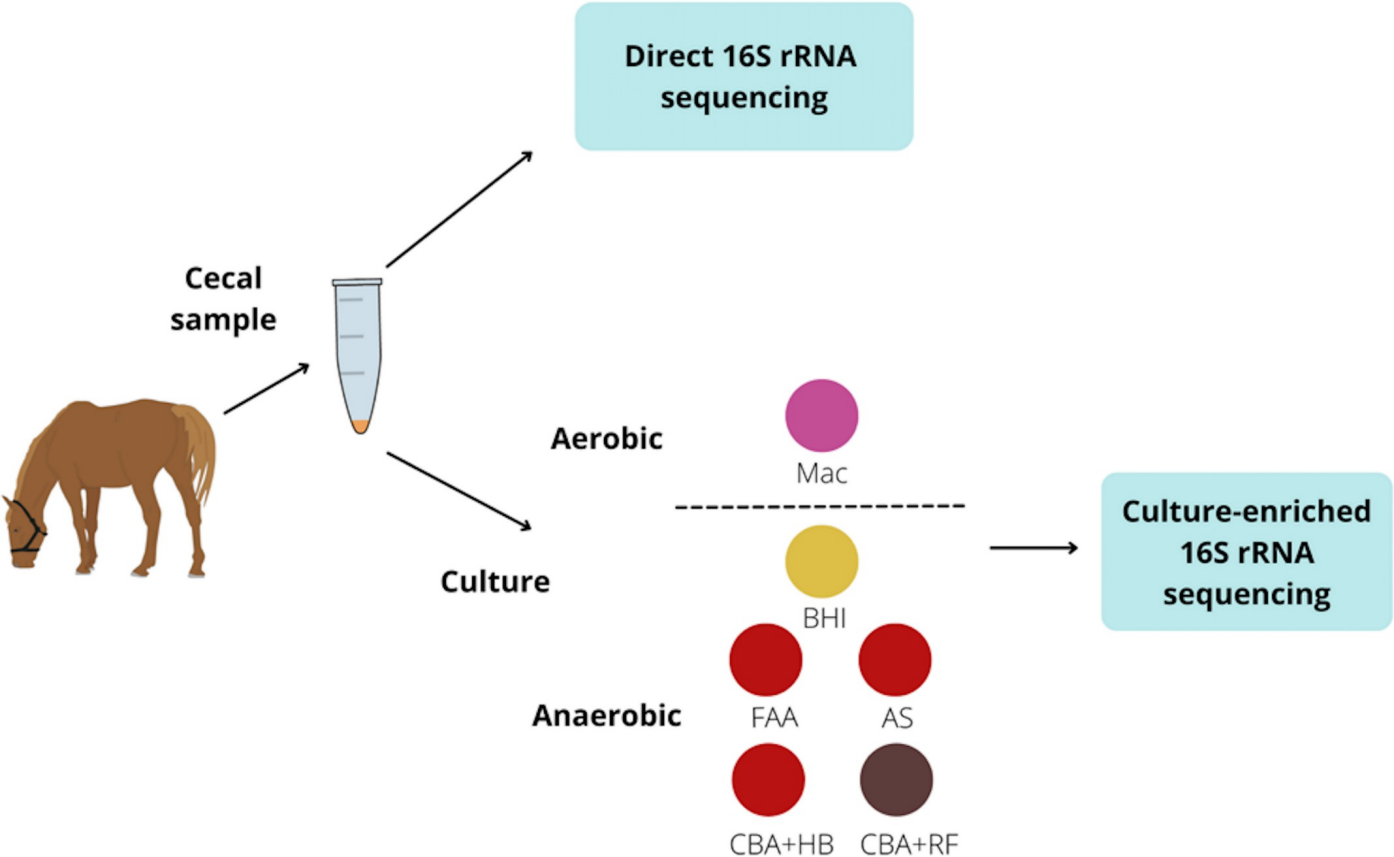
Culture-enriched 16S rRNA gene sequencing workflow. Cecal samples were collected from horses. One aliquot was submitted for direct 16S rRNA sequencing. Other aliquots were plated onto 6 different media types and incubated anaerobically and anaerobically. Colonies were harvested and submitted for culture-enriched 16S rRNA sequencing. Abbreviations: MacConkey agar (Mac), fastidious agar with horse’s blood added (FAA), Columbia agar with horse’s blood added (CBA+HB), Columbia agar with rumen fluid added (CBA+RF), BHI3 with carbohydrate added (BHI), and AS (samples plated on CBA+HB after alcohol shock).

All the described procedures above were performed in an anaerobic chamber (BactronEZ, Sheldon Manufacturing Inc.) with the following gas composition: 10% carbon dioxide, 5% hydrogen and 85% nitrogen. All the culture medias, except for MacConkey agar were incubated at 37°C anaerobically. The MacConkey agar was incubated under aerobic conditions at 37°C. After 72 hours, 3 ml of BHI with 0.1 mM of L-cysteine hydrochloride were added to each plate, and the colonies were harvested. Then, 300 μL were placed into a tube containing 1 ml of GES and 1 mL was placed in a cryotube containing 0.5 mL of glycerol 75%. All samples were stored at -20°C until DNA processing (Fig 1).

### DNA isolation, 16S rRNA Illumina sequencing and analysis

Bacterial colonies growing on each culture medium type were harvested, growth from both dilutions were combined for DNA extraction. Genomic DNA was extracted as previously described [13] with minor modifications. Samples were transferred to screw cap tubes containing 2.8 mm ceramic beads, 0.1 mm glass beads, guanidium thiocyanate, ethylenediaminetetraacetic acid (EDTA) and Sarkosyl solution (GES) and sodium phosphate buffer as described. Samples were bead beat and centrifuged as described and the supernatant was further processed using the MagMAX Express 96-Deep Well Magnetic Particle Processor from Applied Biosystems with the Multi-Sample kit (Life Technologies#4413022). Purified DNA was used to amplify the V3-V4 region of the 16S rRNA gene by PCR. 50 ng of DNA was used as template with 1U of Taq, 1x buffer, 1.5 mM MgCl2, 0.4 mg/mL BSA, 0.2 mM dNTPs, and 5 pmoles each of 341F (CCTACGGGNGGCWGCAG) and 806R (GGACTACNVGGGTWTCTAAT) Illumina adapted primers, as described previously [14]. The reaction was carried out at 94°C for 5 minutes, 5 cycles of 94°C for 30 seconds, 47°C for 30 seconds and 72°C for 40 seconds, followed by 25 cycles of 94°C for 30 seconds, 50°C for 30 seconds and 72°C for 40 seconds, with a final extension of 72°C for 10 minutes. Resulting PCR products were visualized on a 1.5% agarose gel. Positive samples were normalized using the SequalPrep normalization kit (ThermoFisher#A1051001) and sequenced on the Illumina MiSeq platform at the McMaster Genomics Facility. Reads were processed using DADA2 [15]. First, Cutadapt was used to filter and trim adapter sequences and PCR primers from the raw reads with a minimum average quality score of 30 and a minimum read length of 100 bp [16]. Sequence variants were then resolved from the trimmedraw reads using DADA2, an accurate sample inference pipeline from 16s amplicon data. DNA sequence reads were filtered and trimmed based on the quality of the reads for each Illumina run separately, error rates were learned, and sequence variants were determined by DADA2. Sequence variant tables were merged to combine all information from separate Illumina runs. Bimeras were removed and taxonomy was assigned using the SILVA database version 1.3.8.

The data was analyzed in R v. 3.6.3. Diversity analysis was conducted after rarefying sequencing depth to 15,000 reads per sample. Observed amplicon sequence variant (ASV) richness and Shannon’s index were used to calculate species richness and evenness, and pairwise differences between diarrhea status were calculated using analysis of variance (ANOVA) with post-hoc Tukey’s Honest Significant Differences. Principal coordinate analysis (PCOa) was used on weighted UniFrac distances to measure the differences in microbial composition between samples. Permutational multivariate analysis of variance (PERMANOVA) was completed to visualize the weighted UniFrac distance [17] matrix with 999 permutations to identify significant differences across sample type and diarrhea status. A maximum likelihood phylogenetic tree was constructed on ASV fasta sequences using the DECIPHER R package v. 2.14 to align sequences before the phangorn R package (v. 2.6.2) was used to generate a tree using the general time reversible with Gamma rate variation model. Phylogenetic ASV content of each sample was visualized using the ggtreeExtra v. 1.3.3. R package. Phylogenetic ASV content of each sample type was visualized using the ggtreeExtra v. 1.3.3 R package. For identifying shared ASV’s and taxa across samples and sample types, a particular taxon or ASV had to be present in a sample at least once. DESeq2 was used to identify differentially abundant genera across diarrhea status for cecum samples. A p-value of 0.05 was considered statistically significant for all analysis except for DESeq2 analysis, which used a significance level of p = 0.01.

## Results

### Horses

The six horses included in the healthy control group (Sample (S) 1–6) had a median age of 18.5 years (range: 3.5–35 years). Four were males and 2 were females. The following breeds were represented, 2 Thoroughbred, 2 Mixed Breed, 1 American Quarter Horse, 1 Oldenburg. The reason for euthanasia was acute or chronic orthopedic issues (n = 5) or aggressive behavior (n = 1). The six horses included in the typhlocolitis group (S 7–12) had a median age of 8 years (range: 3–20 years). The age was not significantly different between these groups (p = 0.20). Four were female and 2 were male. Regarding breed, 2 were Standardbred, 2 Thoroughbred, 1 American Quarter Horse and 1 Haflinger. All horses had confirmed typhlocolitis on histologic examination of the cecal tissue, but an etiological agent was not detected in any of the horses after necropsy examination. Intestinal contents were collected from all cases during necropsy and submitted for anaerobic and aerobic bacterial culture. *Clostridium perfringens* was isolated from 3 horses (S7, S10, S11), but enterotoxin testing was only performed subsequently in one horse and was negative for all tested pathogens (S11). *C*. *perfringens*, *Escherichia coli* and *Clostridioides difficile* were also isolated from a horse (S7), however toxigenicity of the clostridial organisms was not confirmed *in vitro*. *Neorickettsia risticii* PCR from intestinal content was performed on 3 horses (S10. S11, S12), which were all negative. Coronavirus PCR was performed only in 1 horse (S8), which was negative. No *premortem* confirmed diagnosis was reached in any case.

### Microbiota composition

At the start of analysis, a total of 81,147 (SD = 24,344) reads per sample (n = 83) were available. Samples with less than 1000 reads (n = 1) were removed for downstream analysis. Following the removal of reads classified as chloroplast and mitochondria, and limiting reads classified to the bacterial kingdom, the samples that remained (n = 82) had an average of 82,124 reads (SD = 22,795).

### Alpha diversity

**Fig 2:**
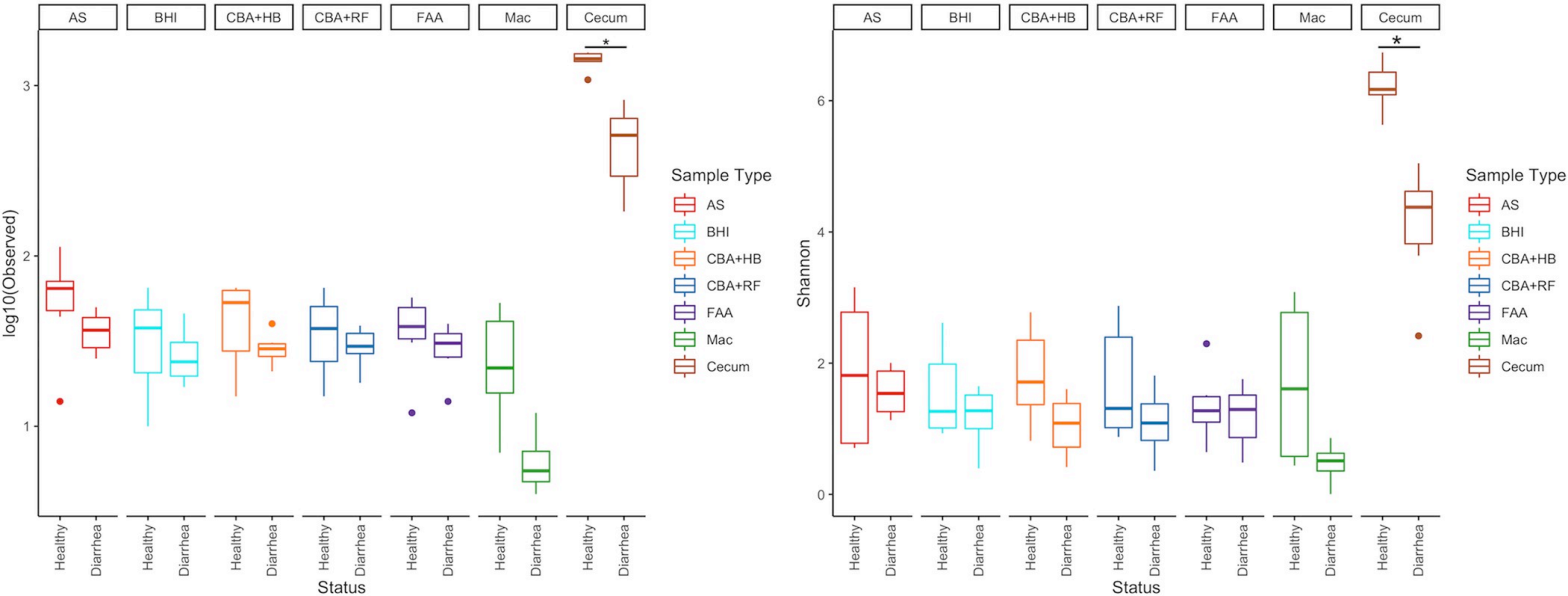
Observed and Shannon diversity of different media types divided by the groups of healthy horses and horses with diarrhea. Abbreviations: MacConkey agar (Mac), fastidious agar with horse’s blood added (FAA), Columbia agar with horse’s blood added (CBA+HB), Columbia agar with rumen fluid added (CBA+RF), BHI3 with carbohydrate added (BHI), AS (samples plated on CBA+HB after alcohol shock), and Cecum (samples sequenced directly, without culture).

**Fig 3:**
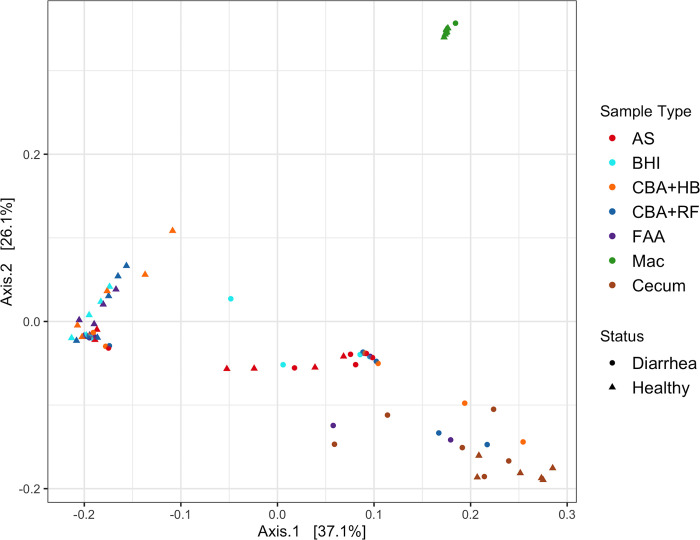
Principal coordinate analysis (PCoA) Plot generated on weighted UniFrac distances of cecal samples from healthy horses and horses with acute colitis either sequenced directly (cecum) or after selective media culture (Mac, FAA, CBA+HB, CBA+RF, BHI and AS). Abbreviations: MacConkey agar (Mac), fastidious agar with horse’s blood added (FAA), Columbia agar with horse’s blood added (CBA+HB), Columbia agar with rumen fluid added (CBA+RF), BHI3 with carbohydrate added (BHI), AS (samples plated on CBA+HB after alcohol shock), and Cecum (samples sequenced directly, without culture).

Diarrheic horses had a lower richness compared to healthy horses, when comparing all 6 media types, as well as the samples sequenced directly (Fig 2). Shannon index was higher in samples directly sequenced than in samples that underwent culture (Fig 3), as expected since each medium condition enriched only part of the community. In addition, when analyzing only cecal samples, diversity was significantly lower (P < 0.05) in diarrheic than in healthy horses (Fig 2).

### Beta diversity

PERMANOVA on weighted UniFrac distances identified significant differences (p < 0.001) in microbial composition between healthy and diarrheic horses for cecum and cultured samples (Fig 3). The PCoA plot based on weighted UniFrac distances showed that cecum samples clustered separately from those that underwent alcohol shock, cultured aerobically on MacConkey agar, or cultured on BHI and FAA. Visually, the microbial composition of the MacConkey samples is substantially different from that of samples cultured under other conditions and media. Both sample type (i.e. type of culture or direct sequencing) and status (i.e., healthy or diarrhea) influence the clustering of the samples, but sample type had a stronger effect (R^2^ = 0.49) on the clustering than did status (R^2^ = 0.06).

### Relative abundance

#### Phylum level

**Fig 4:**
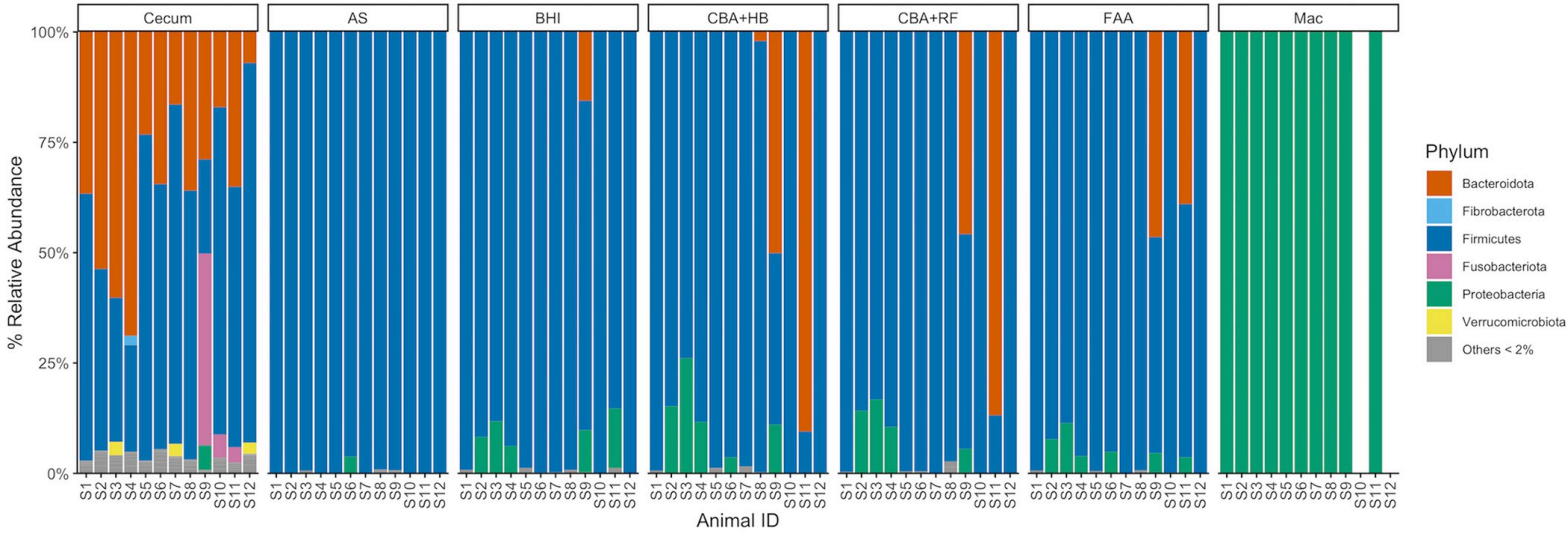
Distribution and relative abundance of bacterial phyla across sample types. Microbiota for each animal ID are partitioned by seven sample types. Bacterial phyla under 2% relative abundance in each sample were grouped. Abbreviations: MacConkey agar (Mac), fastidious agar with horse’s blood added (FAA), Columbia agar with horse’s blood added (CBA+HB), Columbia agar with rumen fluid added (CBA+RF), BHI3 with carbohydrate added (BHI), AS (samples plated on CBA+HB after alcohol shock), and Cecum (samples sequenced directly, without culture). Healthy horses: S1-S6. Acute typhlocolitis horses: S7-S12.

**Fig 5:**
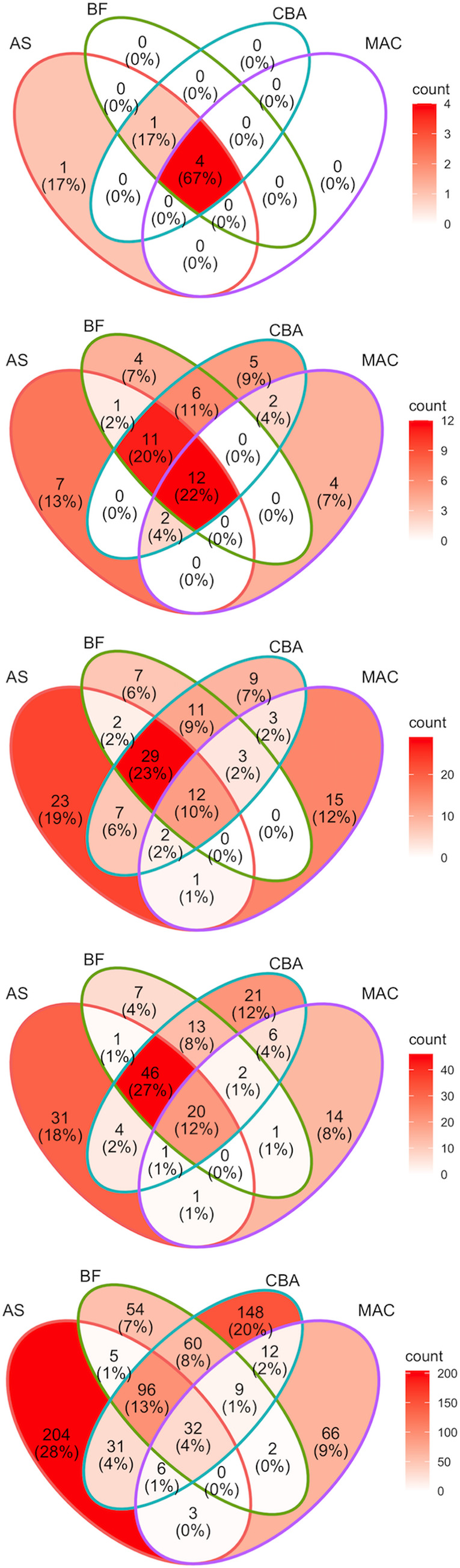
Distribution of culture-derived equine microbiota across different culture media. Venn diagrams display the number of bacterial taxa in common between different culture medias. Microbiota of CBA+HB and CBA+RF (CBA) were grouped together, as well as BHI and FAA (BF), for easier visualization of Venn diagrams. Bacterial taxa were grouped to different taxonomy levels in each individual Venn diagram (from top to bottom: phylum, family, genus, species, individual ASV). Venn diagram sections are shaded based on bacterial taxa count. Abbreviations: MacConkey agar (Mac), fastidious agar with horse’s blood added (FAA), Columbia agar with horse’s blood added (CBA+HB), Columbia agar with rumen fluid added (CBA+RF), BHI3 with carbohydrate added (BHI), AS (samples plated on CBA+HB after alcohol shock), and Cecum (samples sequenced directly, without culture).

Cecal samples from healthy horses contained *Bacteroidetes* and *Firmicutes* predominantly. In addition to these phyla, samples from diarrheic horses contained *Fusobacteriota* (Fig 4). Samples from AS contained only *Firmicutes*, but in one healthy horse a low abundance of *Proteobacteria* was detected. Samples of healthy and diarrheic horses cultured on CBA+HB, CBA+RF and FAA had high relative abundance of Firmicutes and a low relative abundance of *Proteobacteria*. *Bacteroidete*s was detected only in two samples from typhlocolitis horses (S9 and S11) cultured on CBA+HB, CBA+RF and FAA, and only one diarrheic horse had Bacteroidetes when the sample was cultured on BHI (Fig 4). *Fusobacteriota* and *Verucomicrobia* were not identified in any of the CBA+HB, CBA+RF and FAA cultures. The Mac media sample contained only *Proteobacteria*, as expected (Fig 4). Venn diagrams demonstrated that at the phylum level, all the samples share 67% of the phyla (Fig 5).

#### Family level

**Fig 6:**
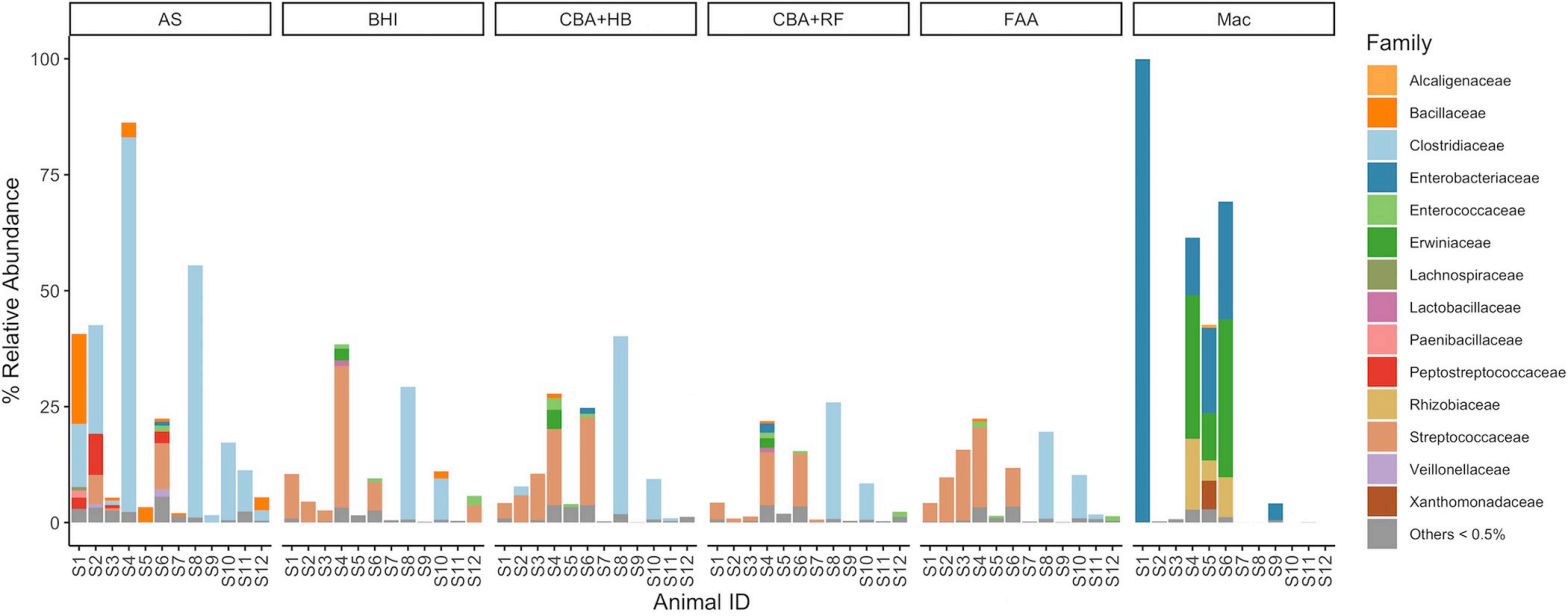
Relative abundance of ASVs in culture-derived samples. Only ASVs that were present in at least one culture-derived sample but absent in all cecum samples are included. Relative abundances were partitioned by culture media and individual animal ID. ASVs were grouped at family taxonomy. Families with relative abundances less than 0.5% were grouped together. Abbreviations: MacConkey agar (Mac), fastidious agar with horse’s blood added (FAA), Columbia agar with horse’s blood added (CBA+HB), Columbia agar with rumen fluid added (CBA+RF), BHI3 with carbohydrate added (BHI), and AS (samples plated on CBA+HB after alcohol shock). Healthy horses: S1-S6. Acute typhlocolitis horses: S7-S12.

At the family level, the family *Clostridiaceae* was the most abundant family detected in AS samples from both healthy and diarrheic horses (Fig 6). Samples from healthy horses cultured on BHI, CBA+HB, CBA+RF and FAA contained high relative abundance of *Streptococcaceae*, but this family was identified in very low relative abundance in samples from diarrheic horses. Two samples of diarrheic horses (S8 and S10) had a higher relative abundance of *Clostridiaceae* than all the other samples when cultured on BHI, CBA+HB, CBA+RF and FAA (Fig 6). *Clostridiaceae* was not present in the samples cultured aerobically on Mac. *Enterobacteriaceae* was the most abundant family isolated from MacConkey agar, followed by *Erwiniaceae* and *Rhizobiaceae*. However, few bacteria were isolated from diarrheic horses using this medium and culture conditions (Fig 6). Venn diagrams demonstrated that FAA and BHI medias shared the most similarities when analyzed at the family level, as well as CBA+HB and CBA+RF (Fig 5).

#### Genus level

**Fig 7:**
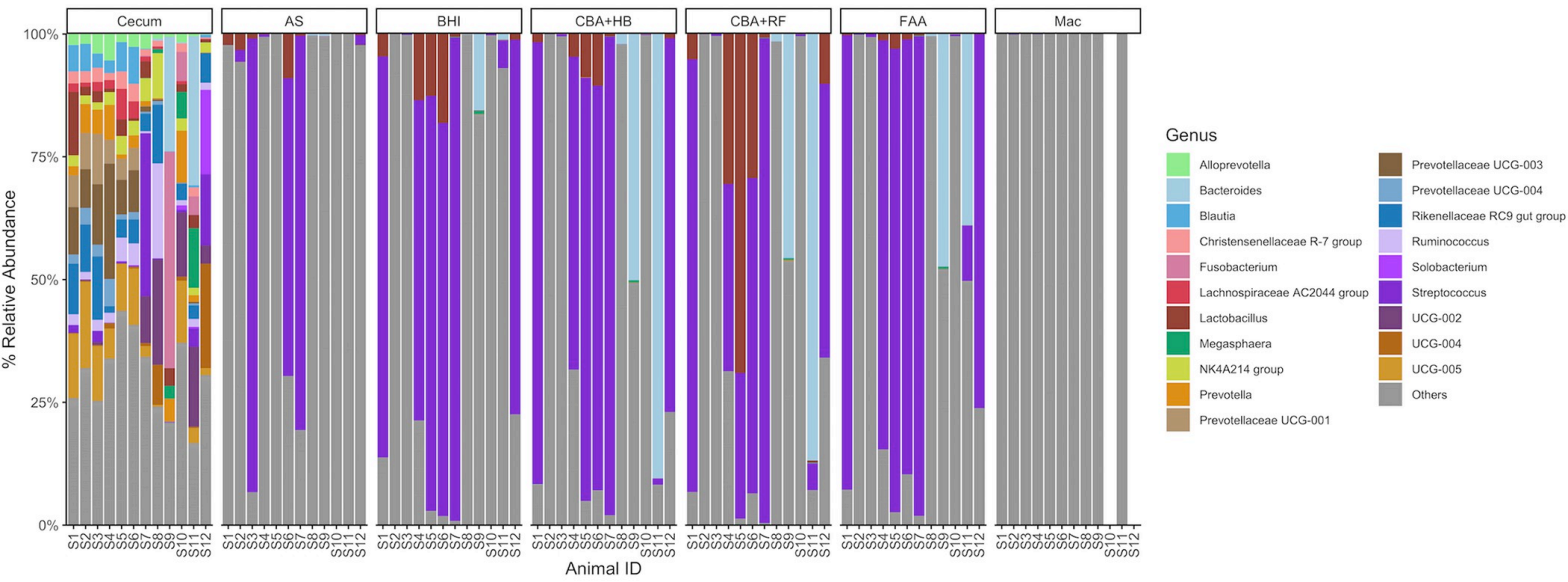
Relative abundance of genus in culture-derived and cecum samples. Abbreviations: MacConkey agar (Mac), fastidious agar with horse’s blood added (FAA), Columbia agar with horse’s blood added (CBA+HB), Columbia agar with rumen fluid added (CBA+RF), BHI3 with carbohydrate added (BHI), AS (samples plated on CBA+HB after alcohol shock), and Cecum (samples sequenced directly, without culture). Healthy horses: S1-S6. Acute typhlocolitis horses: S7-S12.

**Fig 8:**
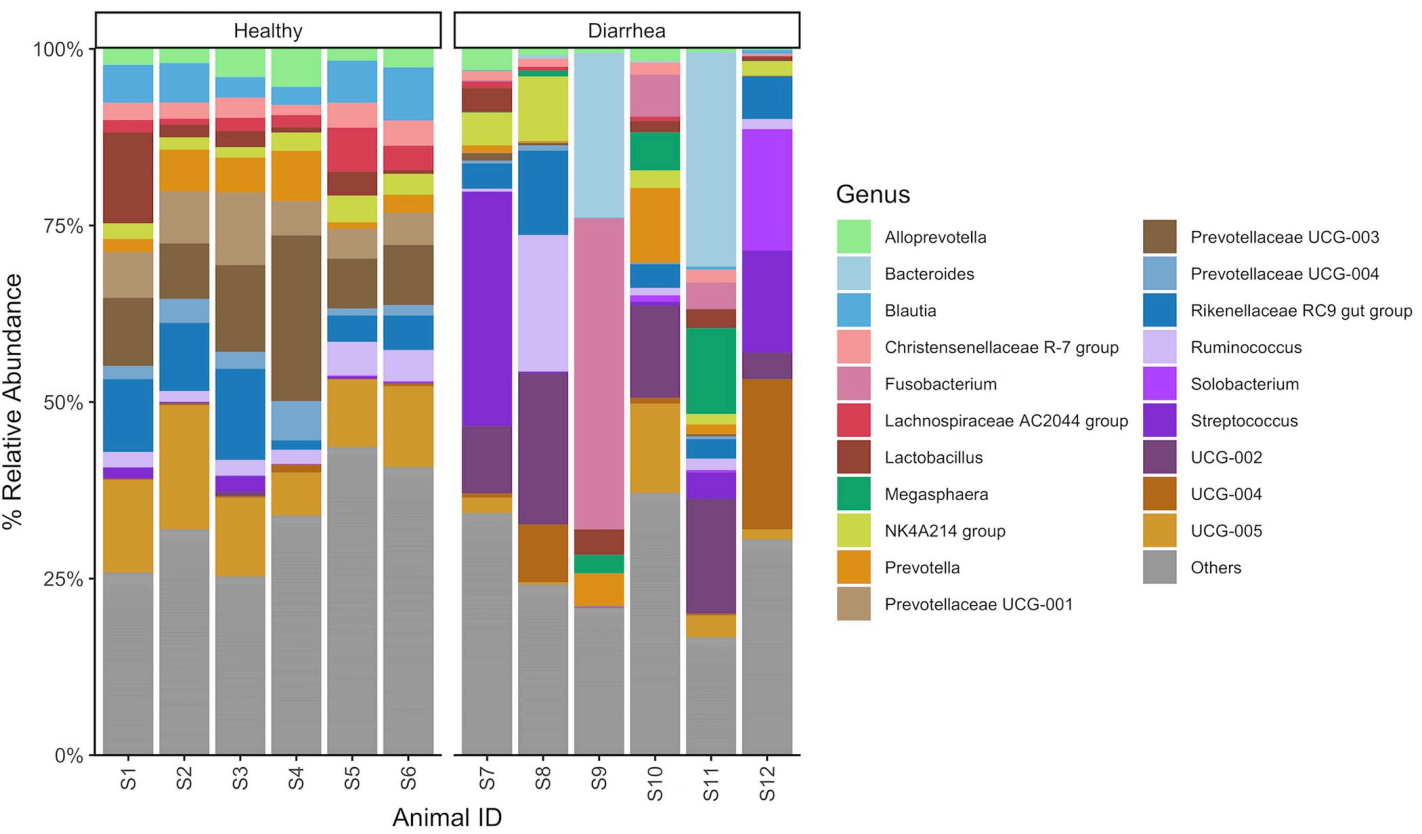
Relative abundance of the top 20 genus in cecal samples of healthy horses and horses with diarrhea.

At the genus level, direct sequencing cecal samples demonstrated more diversity compared to the samples that underwent culture (Fig 7). The anaerobic conditions and media types selected for mostly *Streptococcus*, *Lactobacillus* and *Bacteroides* (Fig 7). Interestingly, *Lactobacillus* was detected mostly in healthy horses while *Bacteroides* was identified in diarrheic ones (Fig 7). When only cecal samples (direct sequencing) were analyzed and separated into healthy and diarrheic horses, it was clear that the microbiota profile differs between these two groups (Fig 8). Healthy animals presented a more uniform profile among the 6 horses and a higher relative abundance of genuses of Prevotellaceae, *Blautia*, Christensenellaceae R-7 group, Lachnospiraceae AC2044 group and *Lactobacillus*, while horses with diarrhea had a more variable population, with *Streptococcus*, *Fusobacterium*, *Bacteroides*, *Megasphaera* and *Solobacterium* being abundant in one or more samples (Fig 8).

**Fig 9:**
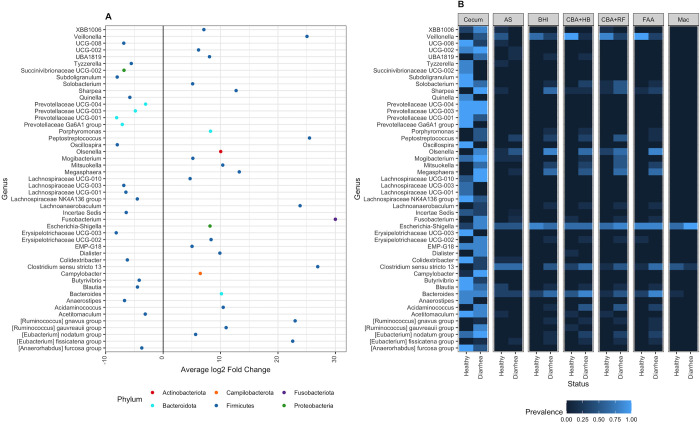
Differential abundance analysis to the cecum genus level bacteria of diarrheic horses showing the difference in abundance of each genus compared to healthy horses (A). The prevalence of significant genera in cecum and cultured samples (B). Abbreviations: MacConkey agar (Mac), fastidious agar with horse’s blood added (FAA), Columbia agar with horse’s blood added (CBA+HB), Columbia agar with rumen fluid added (CBA+RF), BHI3 with carbohydrate added (BHI), AS (samples plated on CBA+HB after alcohol shock), and Cecum (samples sequenced directly, without culture).

The differential relative abundance analysis of cecum genus level bacteria when comparing diarrheic animals and healthy animals showed a significant decrease (p < 0.01) in the abundance of genuses of *Prevotellaceae* and *Lachnospiraceae* in diarrheic animals, while there is a 3-fold average increase in the abundance of *Fusobacterium* and *Clostridium sensu stricto* 13 and a 1-fold average increase in *Olsenella*, *Escherichia-Shigella* and *Bacteroides* in diarrheic compared to healthy horses (Fig 9A). Fig 9B illustrates the most relative abundant genus in each of the sample types divided into healthy and diarrheic horses and demonstrates the different genus profile among sample types.

Venn diagrams demonstrated that at the genus, species and individual ASV, only 10% of genera, 12% of the species and 4% of the ASV, are shared by all the medias (Fig 5). Alcohol shock seems to be the medium that differs the most from the other culture media (Fig 5).

### Phylogenetic tree

**Fig 10:**
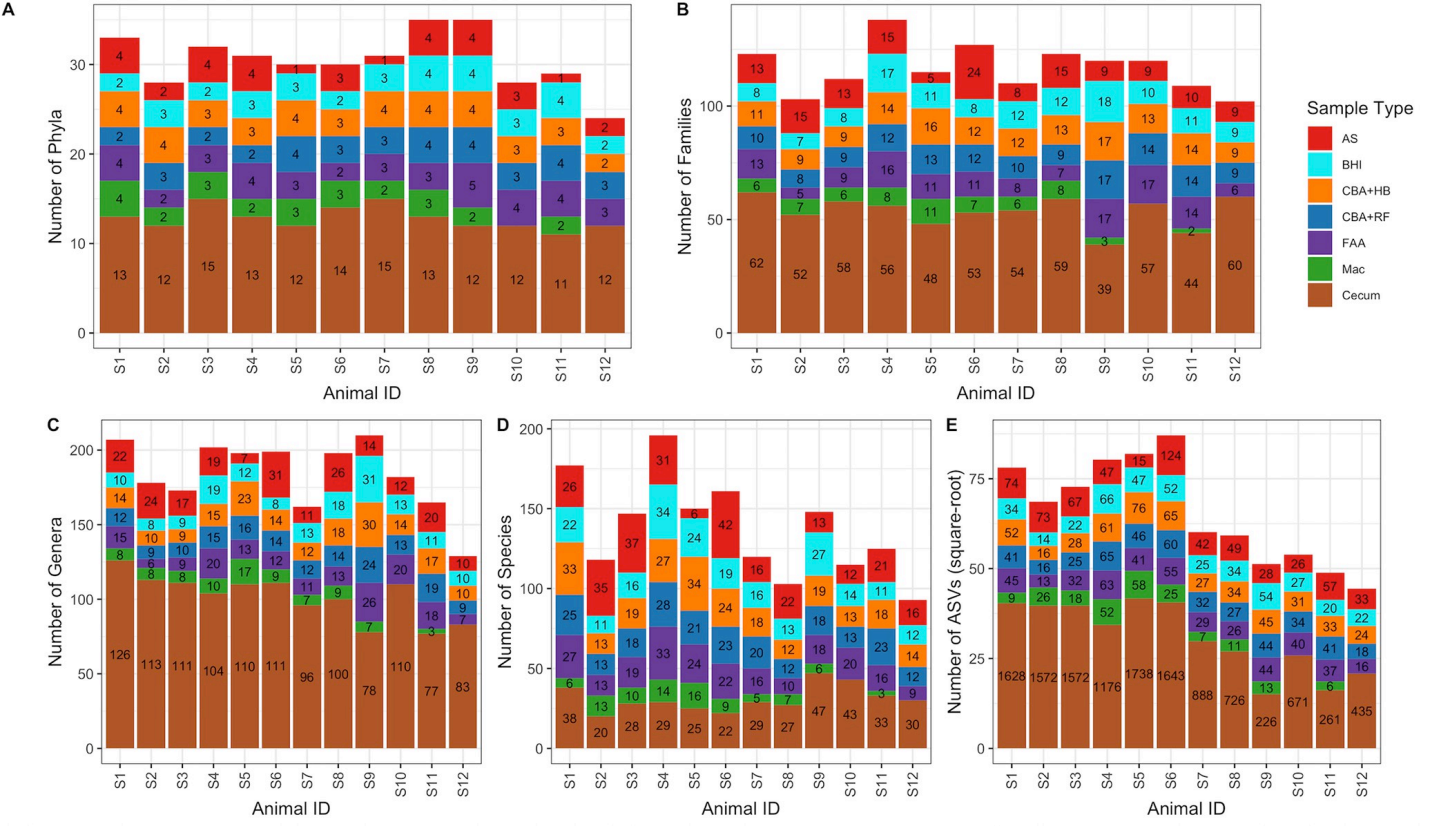
Number of bacterial taxa present in each sample type at multiple taxonomic ranks. The number of unique bacterial taxa are displayed for each sample type at A) phylum, B) family, C) genus, D) species, and E) ASV level. E) The y-axis is square-rooted for easier visualization of major differences between ASV counts between sample type. Unaltered number of ASVs are present within the stacked bars. Abbreviations: MacConkey agar (Mac), fastidious agar with horse’s blood added (FAA), Columbia agar with horse’s blood added (CBA+HB), Columbia agar with rumen fluid added (CBA+RF), BHI3 with carbohydrate added (BHI), AS (samples plated on CBA+HB after alcohol shock), and Cecum (samples sequenced directly, without culture). Healthy horses: S1-S6. Acute typhlocolitis horses: S7-S12.

The number of taxa identified at the different taxonomic level (e.g., phyla, family, genera, species and ASVs) was higher in samples sequenced directly from cecal content than in samples that underwent targeted culture (Fig 10). The number of ASVs identified in diarrheic horses was lower than the one identified in healthy ones (Fig 10).

**Fig 11:**
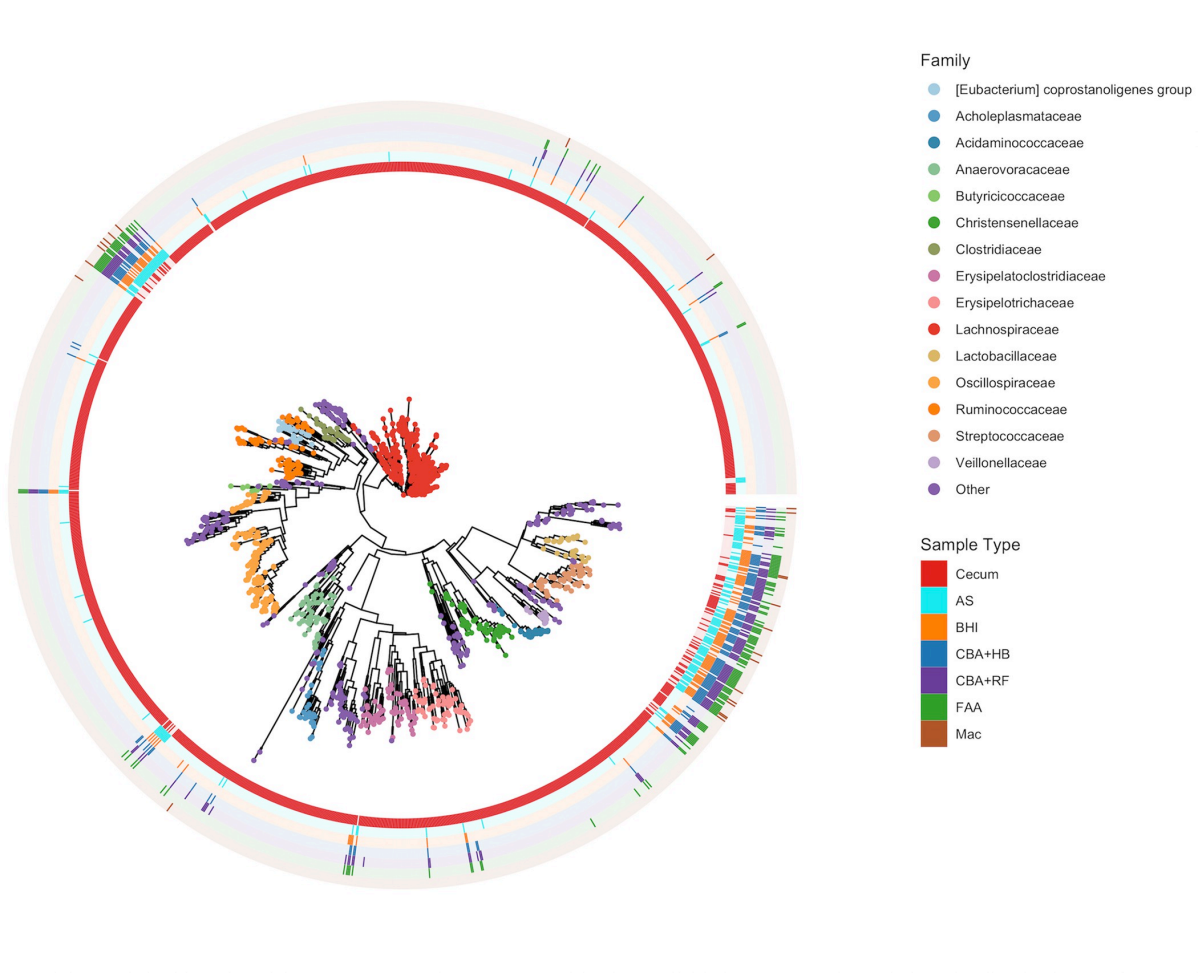
Phylogenetic tree of ASV distribution across each sample type. Maximum likelihood tree tips are coloured by family-level taxonomic classification. Tips were labeled for ASVs that were present in multiple samples, and if species-level taxonomy was available. Colored tiles represent ASVs being present in at least one sample of each sample type. Stacked bar plots on outer ring demonstrate the relative abundance of each ASV and are colored by sample type. Abbreviations: MacConkey agar (Mac), fastidious agar with horse’s blood added (FAA), Columbia agar with horse’s blood added (CBA+HB), Columbia agar with rumen fluid added (CBA+RF), BHI3 with carbohydrate added (BHI), AS (samples plated on CBA+HB after alcohol shock), and Cecum (samples sequenced directly, without culture).

A phylogenetic tree of the phylum *Firmicutes* was created (Fig 11). This tree illustrates that most families were only detected in cecal samples (direct sequencing), for example *Lachnospiraceae*, *Oscillospiraceae*, *Christensenellaceae* (Fig 11). A few families were mostly detected on samples after culture, such as *Streptococcaceae* and *Lactobacillaceae* (Fig 11).

## Discussion

This study compared direct sequencing and sequencing after targeted bacterial culture of the GIT microbiota of intestinal contents of horses with and without acute typhlocolitis. As reported in previous studies [11, 12, 18], the alpha diversity decreases in horses with acute typhlocolitis, and the taxa profile is different. Whether this shift of diversity profile is the cause or a consequence of the typhlocolitis remains to be determined. Our study showed that there was a decrease in *Lachnospiraceae* and *Prevotella* in diarrheic horses, and an increase in *Bacteroides*, *Fusobacterium* and *Peptostreptococcus*. The role of *Prevotella* [19] and *Bacteroides* [20] have been reviewed and they are both fundamental for the gastrointestinal physiology and fermentation but could also have a potential role as pathobiont (opportunistic bacteria) in humans. This shift in genus relative abundance could lead to alteration in the gastrointestinal fermentation and immunomodulation, and has also been proposed to occur in other animals with diarrhea [21]. In addition, *Bacteroides fragilis* has been speculated to cause diarrhea in foals [22, 23] and its role in adult horse diarrhea has not been explored but warrants investigation.

Previous studies comparing the fecal microbiota of horses with colitis and healthy horses using 16S rRNA sequencing found that *Firmicutes*, *Bacteroidetes* and *Proteobacteria*, in this order, were the dominant phyla in fecal samples of healthy horses. However, a shift to *Bacteroidetes*, *Firmicutes* and *Proteobacteria* occurred in horses with colitis [11, 12]. Moreover, *Fusobacteriota* was also identified to be more prevalent in horses with colitis than in healthy horses [11, 12]. Our study also identified that *Firmicutes* and *Bacteroidetes* were the most prevalent phyla in healthy animals, but interestingly *Bacteroidetes* was more prevalent than *Firmicutes* in 3 healthy individuals. Also, an increase in prevalence of the phylum *Bacteroidetes* was not identified in diarrheic animals in this study, but there was an increase of *Bacteroides* in diarrheic horses. An increase in *Firmicutes*:*Bacteroidetes* ratio has been associated with possible dysbiosis secondary to the administration of doxycycline in horses [24] and these two phyla differences could potentially be correlated with typhlocolitis. Another difference between our study and a previous report [12] is that *Lactobacillus* has been reported to be increased in diarrheic horses, while our study identified a higher abundance of *Lactobacillus* in healthy horses. Although some of these differences could be due to different methodologies, these incongruences between studies highlight the current lack of knowledge of the healthy equine gut microbiota and the role of different taxa on health and disease. Finally, our study also identified a high prevalence of *Firmicutes* in diarrheic horses, suggesting taxa belonging to this phylum could be associated with gastrointestinal inflammation or the disease conditions facilitate their overgrowth. Specifically, *Clostridium* sensu stricto increased in diarrheic horses by 3-fold compared to healthy horses. Of interest, *C*. *perfringens* (*Clostridium* sensu stricto genus) was cultured from 3 horses from the diarrhea group which is consistent with other reports in the literature. However, it is unclear whether *C*. *perfringens* present in diarrheic horses is the cause of diarrhea or its overgrowth during colitis. These results highlight the importance of this genus in health and disease and how essential it is to follow-up positive culture results with ELISA tests to detect toxins to determine association, and possibly causation, between the bacterium and the disease.

There are currently only two studies specifically investigating the gut microbial changes in horses with colitis [11, 12]. Although these reports provide inciteful information, the sole use of direct 16S rRNA gene sequencing can be limiting as it biases the results and obscures bacteria that are present in smaller numbers but could be still pathogenic. Bacterial culture targeting specific bacteria of interest prior to gene sequencing has demonstrated to be efficient in exposing bacteria that would be otherwise obscured by the overwhelming number of other bacteria when the sample is sequenced directly [25]. In our study, we detected significantly less taxa of bacteria from samples that underwent culture than in the samples sequenced directly. However, the less abundant number of taxa and more specific environmental conditions facilitated the detection of bacteria from the family *Clostridiaceae* in AS samples, *Enterobacteriaceae* in MAC samples, as well as the presence of significantly more *Streptococcaceae* in healthy animals than in horses with colitis in BHI, CBA+HB, CBA+RF, and FAA cultures. These differences were not detected in the samples directly sequenced and could represent new opportunities for the deeper understanding of the changes in bacterial microbiota of horses with colitis. More recently, a study evaluated the fecal microbiota of horses through 16S gene sequencing aiming to identify associations of microbiota changes with survival and development of laminitis in horses [26]. This report shows a higher relative abundance of *Enterobacteriaceae*, *Lactobacillus*, *Streptococcus* and *Enterococcus* in horses with acute colitis. Again, our study results differ from the results from Ayoub *et al*. (2022) [26] as we detected more *Lactobacillus* and *Streptococcus* in healthy horses. These differences could be due to the fact that they used fecal samples and we used cecal samples, or that culture allowed us to detect a different GIT microbial profile in healthy horses.

Taxa from the phylum *Fusobacteriota* were only detected in samples sequenced directly. Two previous studies [11, 12] also identified that *Fusobacteriota* increased in horses with diarrhea. The blood agar plates (CBA+HB) cultured anaerobically should have been sufficient for *Fusobacteriota* to grow [27]. One possible reason for the lack of growth of this phylum could be that the samples underwent freezing and thawing cycles prior to culture which negatively affect bacterium viability. Although some bacteria may not have survived to this protocol, their DNA is still detectable, which could explain the lack of growth on the media plates yet adequate molecular detection. Although unlikely, *Fusobacteriota* inhibition growth by other bacteria on CBA should be considered, and therefore, the use of specific *Fusobacterium* Selective agar should be considered for further investigation of this genus.

The phylogenetic tree generated with the data obtained from the 16S rRNA sequencing highlights the targeted characteristic of the culture medias when compared to the sample that was sequenced directly. Our media choices were based on previous published data [11], which allowed us to focus on the specific taxa that may play a role in disease such as *Enterobacteria* and *Clostridia*. Some families of *Firmicutes* were only detected in samples sequenced directly, while others were only detected after culture. This difference highlights the limitations of both techniques and the importance of combining them for more comprehensive studies, as culture can emphasize organisms not detected by 16S profiling. Culture conditions that were more selective (Mac) separated from the cecum samples, while non-selected media (CBA+HB and CBA+RF) were more similar to the cecum samples, illustrating how selective media can dictate which bacteria are detected on consecutive genetic sequencing. Within a few families for example, small changes in ASV were detected in cultured samples when compared to the sample sequenced directly. Whether such changes may result from factors related to bacterial replication *in vitro* or whether the sequences were cleaner (possibly less contamination with host DNA) is unknown.

The cecum was chosen as the site of collection due to its location in the abdominal cavity. The samples were collected immediately after euthanasia to avoid postmortem proliferation of some of the microbes, which would potentially happen until necropsy time. The cecum is fixed in the abdominal cavity and therefore, the samples could be acquired from the same place in all the horses. In addition, although it has been shown that fecal samples can show microbiota changes in horses with colitis [11, 12], we believe that cecum samples could represent changes of horses with typhlocolitis more precisely.

The small number of biological samples is considered an important limitation since this prevented us from comparing large data sets from healthy and diseased animals. Sample size calculations are rare in microbiota studies as in most cases, the expected difference is not a precise number. A sample size calculation was based on previous studies that were able to detect differences between healthy and diseased animals using a small sample size. The fact that this study was indeed able to differentiate healthy and acute typhlocolitis horses demonstrates that the sample size is adequate for most of the comparisons, but not necessarily all. However, there is a significant limitation when extrapolating the data from these few horses to a larger population of sick horses, especially from different areas, and with potential different etiologies. Another limitation of our study is that we used two methodologies (bacterial culture and 16S gene sequencing) that are biased approaches for the analysis of microbiota. Nevertheless, these methods are widely used in the study of gut microbiota in human and veterinary medicine, especially for diseases of which microbiology is not well understood/characterized such as equine acute colitis, and were therefore, chosen for this research. Further, we only used 6 different types of media/conditions in this study, which limits the culture of some bacteria. A study that performs culture with other media, such as Man, Rogosa & Sharpe and Bifidobacteria Selective Media could contribute and add to the specific differences between the GIT microbiota of healthy horses and horses with colitis. The use of unbiased methodologies such as metagenomics could significantly add to the current knowledge of equine microbiota in health and disease in the future, as it not only gives the relative abundance estimates of microbiota, it also estimates the functional profile of the microbiome. In addition, the samples used in the study were local luminal samples, and therefore, pathogens primarily associated with the mucosa could be less represented in the local lumen samples [12]. Lastly, our study focused only on the bacteria community of the GIT. The investigation of parasite and fungal association with acute typhlocolitis in horses has already been initiated [28, 29] and the role of a possible viral community deserves further investigation.

## Conclusion

This study showed that the alpha diversity significantly decreased in cecal contents of horses with acute typhlocolitis. In addition, it demonstrated that the results obtained with target bacterial culture differed significantly from those of direct gene sequencing and provided a more specific and detailed information window about the microbial composition of this compartment. Culture-enriched sequencing is a viable additional approach to better understanding of the equine gastrointestinal microbiota. The potential role of *Fusobacterium* and *Bacteroides* in some cases of acute typhlocolitis was raised and deserves further investigation.
